# Toward Individualized Prediction of Binge-Eating Episodes Based on Ecological Momentary Assessment Data: Item Development and Pilot Study in Patients With Bulimia Nervosa and Binge-Eating Disorder

**DOI:** 10.2196/41513

**Published:** 2023-02-23

**Authors:** Ann-Kathrin Arend, Tim Kaiser, Björn Pannicke, Julia Reichenberger, Silke Naab, Ulrich Voderholzer, Jens Blechert

**Affiliations:** 1 Department of Psychology Centre for Cognitive Neuroscience University of Salzburg Salzburg Austria; 2 Department of Clinical Psychology University of Greifswald Greifswald Germany; 3 Schoen Clinic Roseneck Prien am Chiemsee Germany; 4 Department of Psychiatry and Psychotherapy University Hospital Ludwig Maximilian University of Munich Munich Germany; 5 Department of Psychiatry and Psychotherapy University Hospital of Freiburg Freiburg Germany

**Keywords:** idiographic, individualized, N of 1, Ecological Momentary Assessment (EMA), Just-In-Time Adaptive Intervention (JITAI), binge eating, literature research, focus group, prediction algorithm, machine learning, Best Items Scales that are Cross-validated, Unit-weighted, Informative and Transparent, BISCUIT

## Abstract

**Background:**

Prevention of binge eating through just-in-time mobile interventions requires the prediction of respective high-risk times, for example, through preceding affective states or associated contexts. However, these factors and states are highly idiographic; thus, prediction models based on averages across individuals often fail.

**Objective:**

We developed an idiographic, within-individual binge-eating prediction approach based on ecological momentary assessment (EMA) data.

**Methods:**

We first derived a novel EMA-item set that covers a broad set of potential idiographic binge-eating antecedents from literature and an eating disorder focus group (n=11). The final EMA-item set (6 prompts per day for 14 days) was assessed in female patients with bulimia nervosa or binge-eating disorder. We used a correlation-based machine learning approach (Best Items Scale that is Cross-validated, Unit-weighted, Informative, and Transparent) to select parsimonious, idiographic item subsets and predict binge-eating occurrence from EMA data (32 items assessing antecedent contextual and affective states and 12 time-derived predictors).

**Results:**

On average 67.3 (SD 13.4; range 43-84) EMA observations were analyzed within participants (n=13). The derived item subsets predicted binge-eating episodes with high accuracy on average (mean area under the curve 0.80, SD 0.15; mean 95% CI 0.63-0.95; mean specificity 0.87, SD 0.08; mean sensitivity 0.79, SD 0.19; mean maximum reliability of *r*_D_ 0.40, SD 0.13; and mean *r*_CV_ 0.13, SD 0.31). Across patients, highly heterogeneous predictor sets of varying sizes (mean 7.31, SD 1.49; range 5-9 predictors) were chosen for the respective best prediction models.

**Conclusions:**

Predicting binge-eating episodes from psychological and contextual states seems feasible and accurate, but the predictor sets are highly idiographic. This has practical implications for mobile health and just-in-time adaptive interventions. Furthermore, current theories around binge eating need to account for this high between-person variability and broaden the scope of potential antecedent factors. Ultimately, a radical shift from purely nomothetic models to idiographic prediction models and theories is required.

## Introduction

### Binge Eating

Binge eating (objectively excessive food intake accompanied by feelings of loss of control) represents a core symptom of bulimia nervosa (BN), binge-eating disorder (BED), and the binge-purge subtype of anorexia nervosa. It is also the most debilitating symptom in most eating disorders (alongside the associated compensatory behavior in BN and binge-purge subtype of anorexia nervosa), accounting for gastrointestinal comorbidities, along with psychological consequences (eg, shame, secrecy, and social isolation [1]). Thus, interventions have focused on binge eating to ameliorate psychological consequences and subsequent purging behavior, which further contributes to oral and dental harms. However, treatment as usual–cognitive behavioral therapy for eating disorders (EDs) is effective for only about 65% of individuals with an ED [2] and has high relapse rates (26.8% across EDs) [3].

### Nomothetic Binge-Eating Models

To predict binge eating, researchers typically rely on *nomothetic theories*—theories that are based on the average characteristics of multiple individuals in groups. Some nomothetic findings hold that individuals with BN and BED overeat in response to negative emotions, whereas healthy controls do not [4]. However, although particular efforts have been directed at predicting high-risk states for binge eating based on a variety of measures (eg, negative emotions or irregular eating patterns) [5,6], nomothetic binge-eating models often fail to translate to an idiographic–*individual*–level [7-10]. To illustrate, nomothetic theories claiming that emotional eating underlies binge eating [11] imply that emotion regulation interventions provide causative help [12,13]. However, this reasoning might not be applicable to patients who are prone to binge eating when impulsive, after extensive fasting periods, or experiencing dissociative states [6,13,14]. Correspondingly, various nomothetic theories of binge eating have proliferated. They differ substantially in the assumed causal mechanisms, which include, but are not limited to, emotional eating, impulsivity, restrained eating, food addiction, ego depletion, associative learning, and emotion-regulation or coping with emotions [4,15-21].

### Idiographic Binge-Eating Models and Interventions

As binge eating can be highly impulsive, automatic, and difficult to resist, interventions that target binge eating based on its antecedents are promising as they attempt to stop the process as soon as possible, before the binge-eating pressure builds up. As such states can fluctuate quickly, they need to be assessed and evaluated with a high timely resolution to inform about the appropriate timing for interventions for high-risk states. Recently, the methodologies of just-in-time adaptive interventions (JITAIs) [22] and high-frequency ecological momentary assessment (EMA) have merged into a methodological framework that can be applied to binge-eating prediction and prevention. JITAIs have been shown to enhance cognitive behavioral therapy in BED and BN [23] and have been successfully implemented in other domains of eating behavior (eg, in weight loss) [24].

In the “OnTrack” weight-loss intervention, Forman et al [24] sampled emotions and stress, next to eating history and context conditions such as watching television or alcohol consumption. By investigating a wide range of antecedents for dietary lapses, they go well beyond what emotional-eating theory suggests as predictors (eg, negative emotions). Similarly, with their “Think Slim” app, Spanakis et al [25] showed that a sample of participants with normal weight and overweight can be clustered into multiple groups according to the different momentary states in which they tend to eat unhealthily. Therefore, applying a single nomothetic binge-eating theory might be insufficient to identify a broad spectrum of individually varying antecedents and would yield inaccurate predictions of binge eating in most individuals [26]. Instead, to cover all relevant antecedents for many patients, a broad set of EMA items is required. Notably, items that serve this purpose in weight disorders (eg, “OnTrack” JITAI-enhanced weight-loss intervention by Forman et al [24]) may not cover all antecedent states that arise in patients with clinical binge eating. Furthermore, despite being often disregarded within nomothetic frameworks, protective factors (eg, positive emotions or healthy coping [27,28]) have the potential to improve prediction accuracies in idiographic machine learning frameworks because of their negative associations with binge-eating likelihood. However, to balance participant burden with broad sampling, a baseline phase with the full item set could be followed by a phase with a reduced EMA-item set, based on a prediction model that identifies the ideographic subset of items that best predict binge eating for a given individual.

### Aims and Hypothesis

This study examined the feasibility of the first part of this approach, that is, whether subsets of items could be found with good prediction accuracies for binge eating.

Furthermore, 2 studies were conducted to establish a conceptual and empirical foundation for JITAIs on binge eating. In study 1, we collected a comprehensive set of binge-eating antecedents in the form of EMA items. We combined a literature review with qualitative and quantitative interviews (focus group with 11 inpatients) following Soyster and Fisher [29]. In study 2, an algorithm was used to select idiographic subsets of binge-eating predictors based on Elleman et al [30], Kaiser et al [10], and Soyster et al [31]. We hypothesized that these idiographic binge-eating antecedents would predict binge eating with high accuracy. This selection and prediction were tested in 13 patients with BN or BED.

## Methods

### Ethics Approval

All participants signed an informed consent form (stating which data were stored, where and for how long, who the investigator was, and the purpose of the study) approved by the ethics committee of the University of Salzburg (EK-GZ: 37/2018).

### Study 1—Development of the EMA-Item Set

#### Literature Research

A PhD-level researcher systematically searched Google Scholar, PsycINFO, and PubMed databases for articles with the word “binge” in their title and the terms “ecological momentary” or “experience sampling” to find risk state descriptors with relevance to binge eating in the literature. The search resulted in 509 articles that were deduplicated and scanned for relevance. Only empirical articles reporting the results of EMA studies on binge eating were retained. A total of 262 articles were subsequently analyzed (see Multimedia Appendix 1, Figure S1 for an attrition diagram).

#### Text Analysis Using Word Embedding

Abstracts of all articles in the literature were retrieved. The R package *text2vec* [32] was used to perform global vector word-embedding analysis on these abstracts. Word embedding is an “unsupervised” learning algorithm that maps words to a vector space based on their similarity. It is unsupervised as no labeling of training data is needed because training is performed on aggregated global word-word cooccurrence statistics from a corpus. A matrix is calculated where each element *X_ij_* represents how often word*_i_* appears in the context of word*_j_* (ie, in the same sentence). Thus, words can be represented numerically and their similarities can be compared [32].

The following parameters were set for training the word vectors (vector dimensions=100, window size=15, and minimum word count to be included in the model=5). The English stop words were removed. Single words (eg, “sadness”), as well as combinations of 2 words (eg, “negative affect”), were allowed in the model. The cosine similarity between word vectors was used to quantify the similarity between word embeddings. This metric computes the angle between 2 vectors to quantify the similarity in the vector space they inhabit. The interpretation of cosine similarity resembles that of the correlation coefficients. Perfectly similar word vectors have a cosine similarity of 1, whereas perfectly dissimilar vectors have a similarity of −1. We calculated the cosine similarity of all retained words with the words “binge” or “binges” retaining only words that had at least a cosine similarity of +.10 or −.10 (resembling a small effect according to the criteria of Cohen [33] for the interpretation of correlation coefficients). In this way, we intended to find words that were conceptually similar to “binge eating” while covering a wide range of binge-eating antecedents.

#### Integration Into a Preliminary Item List

In the next step, 2 authors independently rated whether a given retained word was quantifiable with a psychometric item (ie, the words “dissociation” or “dissociative” were rated as quantifiable with the item “I feel detached from myself.” [0=*not at all* to *very much*=100]) and in terms of usefulness for an EMA survey. Items were only retained if they were rated as quantifiable and useful by both authors. Overlapping constructs were organized into categories to reduce redundancy. Finally, a preliminary list of 47 items was compiled from the empirical and theoretical constructs and complemented by constructs derived from previous EMA studies (Multimedia Appendix 2, Table S1).

#### Patient Focus Group

A focus group of inpatients (11 female adolescents and young adults in treatment for regular binge-eating episodes at the Schoen Clinic Roseneck, Germany) complemented this literature-based approach. It was conducted to tap into antecedents that nomothetic EMA research might have overlooked so far. After an individual written brainstorming session on “triggers and circumstances associated with binge eating,” the inpatients rated the preliminary list of EMA items on relevance to their binge-eating episodes (“happens before/during/after binge eating*…*”: 1=*[almost] never*, 3=*might or might not*, 5=*[almost] always*). A moderated discussion of the brainstormed and provided items concluded the sessions.

Next, 2 researchers analyzed the rating data and integrated patient-generated items. This led to the following changes: several constructs missing in the preliminary item list were identified and items were added to cover these gaps (eg, eating based on internal opposed to external motivation: “Did you eat on your own accord?”; (not) following a regular meal structure: *“*How much did you follow a regular meal structure today?*”*; and restricting specific foods: “Are you restricting on certain foods right now?”).

The focus group participants further rated 27 of the provided items as positively associated with their binge-eating episodes (mean >3.5), 11 items as negatively associated (mean <2.5), and 9 items as unrelated to their binge-eating episodes (mean 2.5-3.5; Multimedia Appendix 2, Table S1). Some items were scored as unrelated (eg, “Right now I feel: tired” and “I engaged in increased levels of sport.”), and items with large SDs (SD >1.00; eg, “Right now I feel: relived,” “Right now I am shopping for groceries.” and “I acted upon my plans regarding my eating behavior.”) were disregarded, merged (eg, “I am in company.” with “I am on my own.”), or exchanged (eg, “I feel strained due to...work / university / school; close social network; wider social network; everyday stressors” with “Do you feel like you can handle all upcoming tasks and problems?”). As the patients expressed concerns over the redundancy of emotional states, 4 more items were disregarded (“Right now I feel: calm/ashamed/guilty/frustrated”). Finally, 4 items regarding eating behaviors such as “resistance to food craving” or “restriction” were rephrased to map more accurately on constructs introduced by the focus group (see Multimedia Appendix 3, Figure S1 for all item iterations)

#### Feedback of Clinicians

Finally, clinicians with experience in ED treatment (n=4) provided feedback on the gaps in the included constructs. This feedback was integrated by adding concepts such as accessibility to tasty food, day structure (ie, regular sleep and eating patterns), self-regulation intentions, and eating alone. This feedback further led us to include the autoregressive effect of binge-eating episodes on subsequent binge-eating risk in our models [34].

#### First Pilot

The EMA items were then piloted by 2 authors and 1 female patient with BN (consistent with the Diagnostic Statistical Manual-5 [DSM-5] [1]) to evaluate content, coverage, wording, and participant burden. Piloting revealed that some items needed further changes to map more accurately on the intended constructs: 1 item about adaptive coping strategies was added (“How much did you try to distract yourself from a possible urge to overeat by *healthy* strategies [e.g., relaxation, social activity, mindfulness, etc.]?”) to complement the items on dysfunctional coping and distraction strategies, which were merged into one item (“how much did you try to distract yourself from a possible urge to overeat by unhealthy strategies [eg, alcohol, cigarettes, drugs, self-harm, etc]?”). Two items were rephrased, and 1 item assessing food craving was split up and rephrased to differentiate *food craving*, *overeating*, and *objective binge-eating episodes* (food craving: *“*how strong is your craving for certain foods right now?*”*; overeating: *“*how strong is your urge to overeat right now?*”*; and binge-eating episodes: “how high would you rate your risk for a binge-eating episode right now?”).

The highly compliant participant with BN (all 84 EMA signals answered) reported that the participant burden was too high. Thus, 6 more items were disregarded to shorten the extensive list of items assessing different forms of self-licensing [35,36] and restrictions. Finally, the authors integrated the information gathered in the previous steps (ie, literature review, feedback of the focus group, feedback from clinicians, and feedback of the pilot patient) to make final iterations to the EMA-item set (see Multimedia Appendix 3, Figure S1 for all item iterations, and Multimedia Appendix 4, Tables S1 and S2 for the final EMA-item set).

### Study 2—Idiographic Predictor Selection and Prediction of Binge Eating From EMA Data

#### Participants

Female patients with current BN (n=12) or BED (n=1) were recruited via mail from the waiting list for inpatient treatment of the Schoen Clinic Roseneck, Germany (n=10), and from web-based forums on eating disorders and psychology (n=3; see Multimedia Appendix 5, Figure S1 for a CONSORT [Consolidated Standards of Reporting Trials] flowchart). This study was advertised as a pilot study for a smartphone-based binge-eating intervention. The data were collected between April 2020 and April 2021.

#### Procedure

All participants completed the following study protocol. First, the BN and BED research diagnoses according to DSM-5 [1] were determined via telephone using the Eating Disorder Examination interview [37] and the Structured Clinical Interview for DSM-IV [38]. Both interviews were adapted to the diagnostic criteria of the DSM-5 (eg, 1 binge-eating episode per week for 3 months instead of 2 binges per week for 3 months).

The participants were then introduced to the EMA items and logged into the customized smartphone app *SmartEater.* SmartEater was used during the subsequent EMA phase, in which signal-based EMA questionnaires were inquired up to 84 times per participant (6 signal-contingent prompts per day, in intervals of 2.5 hours for 2 weeks; questionnaires expired 1 hour after the initial prompt). In addition, an event-contingent EMA questionnaire on overeating, loss of control, and binge-eating episodes was accessible. Participants were instructed to fill in this event-contingent questionnaire whenever they felt like they overate or felt a sense of loss of control over food intake or both. The event-contingent questionnaire included questions to differentiate between subjective and objective binge eating and objective overeating (Multimedia Appendix 4, Table S2). EMA items assessing emotions were presented in a randomized order. However, the other items were presented in a fixed order to prevent carryover effects. The participants were able to review and change their answers through a “back” button. Answering all items (except branched items) was mandatory for submission of the questionnaires.

After the EMA phase of 2 weeks, a JITAI phase of 2 weeks started, in which the participants received short intervention suggestions from the app to prevent binge-eating episodes at ideographically predicted high-risk times. Every study stage was accompanied by web-based questionnaires that assessed current eating behavior pathology, demographic data, perceived acceptability, feasibility, and so on. Data from the intervention phase were not covered in the present article. For reimbursement, the participants received €30 (US $32.80) and personalized feedback on their EMA data and psychometric web-based questionnaires.

#### Data Preparation and Measures

To avoid the violation of the assumption of equally spaced time series [39], empty rows were inserted in the data set after every last signal for a given day. This prevented the prediction algorithm from regressing on data from the previous day.

##### Binge-Eating Episodes–Criterion

Objective binge-eating episodes, characterized by (1) “feelings of loss of control over eating behavior” and (2) “consumption of objectively large, inappropriate amounts of food” [1,37,40], were identified from eating episodes reported over the signal-based (1 item: “Was your meal a main meal**,** snack, or binge?”) and event-based EMA questionnaires (2 items: “Would other people rate the amount of food as excessive under similar circumstances?” and “Did you feel like you are losing control of your eating behavior?”). The signal-based and the 2 event-based items were recoded into a binary variable indicating the occurrence of an objective binge-eating episode (binge-eating episode reported=1, no binge-eating episode reported=0). As the algorithm was supposed to predict future binge-eating episodes, this variable was shifted backward in time by one signal (approximately 2.5 hour).

##### EMA Predictors

An unshifted version of the binge-eating variable was included as a possible predictor of the autoregressive effects of binge eating. Furthermore, additional EMA items (n=31) were used to model possible binge-eating antecedents. Thus, only items that were assessed with every signal-based questionnaire were included (aside from the binge-eating classifier), as each variable needed to have a sufficient percentage of data points within a person (see Multimedia Appendix 4, Table S1 for the wording of each item).

##### Time Predictors

Time variables, especially in the form of circles and distinct times of day, have been shown to be highly predictive in everyday life [41]. EMA studies have even found peak times for certain binge-eating antecedents (ie, food cravings or hunger; [42]) and binge eating itself [43-45]. Thus, as temporal data are passively collected in the EMA setting via timestamps, without additional participant input, we decided to include different temporal predictors that could detect a single high-risk time per day (24-hour oscillation) or several times per day (sub-24 hour oscillation).

Variables representing 8-, 12- and 24-hour sinusoidal and cosinusoidal cycles were computed based on the cumulative sum of time differences between assessments (eg, 10:30 AM-8 AM, 1 PM-10:30 AM=2.5, 5, 7.5...). For example, a 24-hour sinusoid cycle was calculated using the following formula: *sin*24*h* = *sin*(2π : 24 * *Δ_t_*), where *∆_t_* is the difference between assessment points in hours (here: 2.5). Finally, dummy-coded variables representing the time of day were calculated for each signal (morning, late morning, early afternoon, afternoon, evening, and late evening). This allows for identifying a daytime when binge eating is particularly likely for a given participant (eg, when returning from work) that could not be well captured by the cyclical predictors.

#### Application of the Best Items Scale that is Cross-validated, Unit-weighted, Informative, and Transparent Algorithm to EMA Data (for Idiographic Predictor Selection and Prediction of Binge Eating)

The machine learning algorithm Best Item Scales that are Cross-validated, Unit-weighted, Informative and Transparent (BISCUIT) [30] of the *bestScales* function from the R package *psych* [46] was applied separately to the EMA data of each patient to select the best idiographic predictors of binge-eating episodes. This method was chosen because of its (1) robustness to missing data; (2) use of unit-weighted scoring of predictors, which was found to be more generalizable, especially in the context of prediction; and (3) tendency to select more parsimonious predictor sets compared with other approaches such as Elastic Net regression [30,47,48]. BISCUIT is a simple algorithm that correlates a set of predictors (here all EMA and time variables) with a criterion (here, the binary time-shifted binge-eating variable at t+1) and retains the predictors with the highest correlation to form a unit-weighted scale [10,30,46]. This scale was then used to estimate the out-of-sample predictive performance using 10-fold cross-validation. The average correlation of the scale with the criterion across 10 cross-validation splits was then computed, and the set of items with the highest cross-validated correlation was retained [30,46]. The output of BISCUIT is the selection of items showing maximum predictive validity, as the cutoff values that lead to the highest combination of sensitivity and specificity are retained [30,46].

Thus, multiple Rs (pairwise Pearson correlations) of all predictors with time-shifted binge-eating episodes (at t+1) were calculated for each participant separately to select the idiographic predictor sets. Furthermore, the area under the curve (AUC) with a bootstrapped 95% CI, specificity, sensitivity, and within- and out-of-sample reliability were calculated as prediction accuracy measures of the idiographic predictor sets and their prediction of binge eating in the next 2.5 hours (t+1).

## Results

### Study 1—EMA-Item Set

The final signal-contingent EMA questionnaire included 36 EMA items (momentary emotions, stress, exhaustion, and context; eg, being alone, social interactions, dissociations, eating behavior, resistance to food craving, distraction, and coping), which were designed to be assessed 6 times per day. In addition, an optional event-contingent EMA questionnaire on overeating, loss-of-control eating, and binge eating was self-initialized and included 20 items. See Multimedia Appendix 4, Tables S1 and S2 for all interval- and event-contingent items and their wording. A flowchart of all iterations applied to the EMA-item set can be found in Multimedia Appendix 3, Figure S1).

### Study 2—Idiographic Predictor Selection and Prediction of Binge Eating From EMA Data (by Application of the BISCUIT Algorithm)

#### Selection of Idiographic Predictor Subsets

The patients (n=13) answered on an average 67.3 out of 84 EMA prompts (SD 13.4; range 43-84; see Multimedia Appendix 6, Table S1 for EMA compliance and occurrences of binge-eating episodes per patient). Across participants, the algorithm selected highly heterogeneous predictor sets of varying sizes (mean 7.31, SD 1.49; range 5-9 predictors) for the prediction of binge-eating episodes.

**Figure 1:**
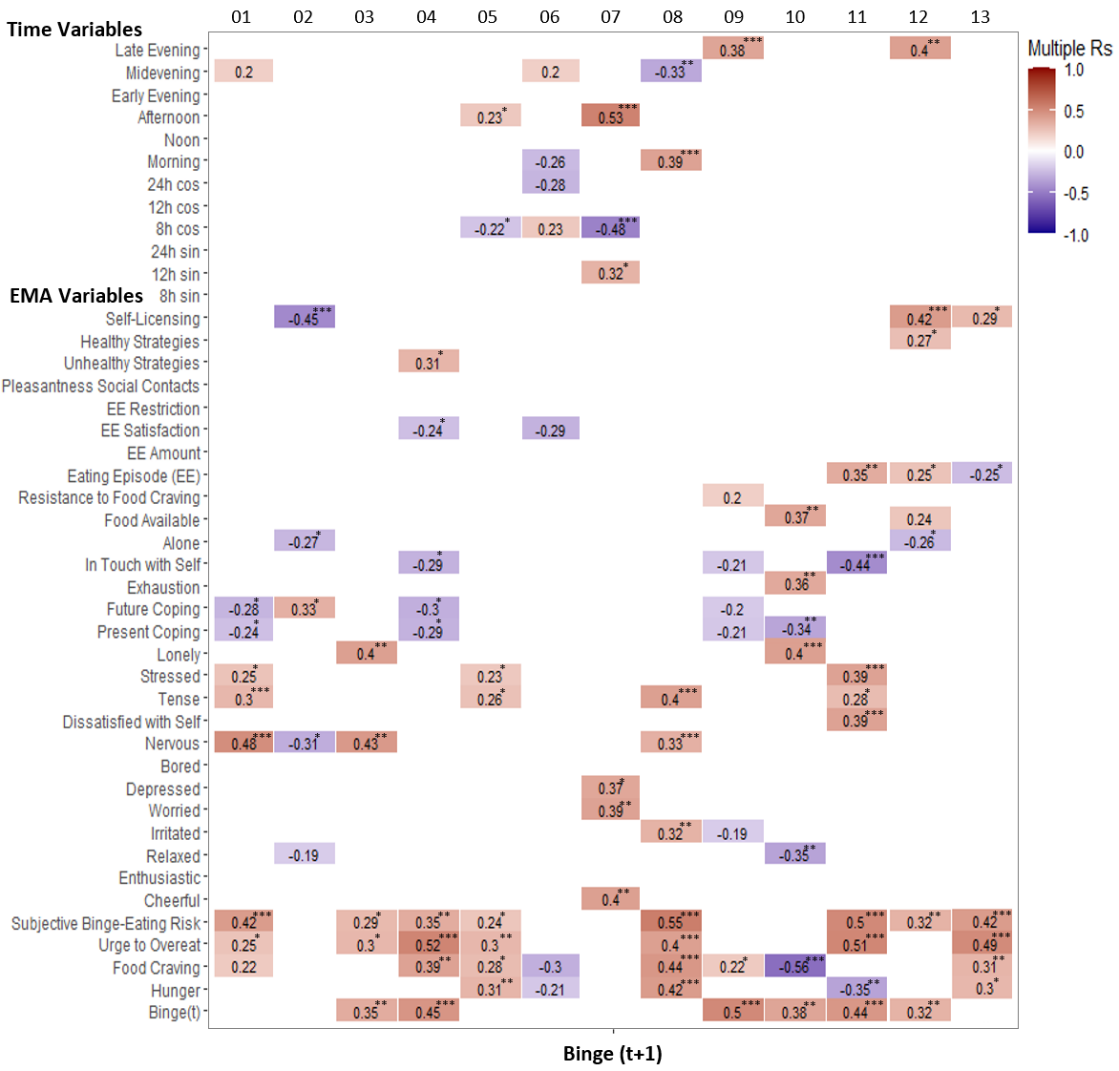
Idiographic predictor subsets for binge eating with Pairwise Pearson Correlations (Multiple Rs) of each selected predictor of binge eating in the next 2.5 hours (t+1). *, **, and *** indicate that the correlations are significant at a level of .05, .01, and .001, respectively; 2-tailed. EMA: ecologic momentary assessment.

Figure 1 shows the idiographic predictor selection that showed maximum predictive validity for each participant. Thus, the predictors (at t) with the highest multiple Rs (pairwise Pearson correlations) with time-shifted binge-eating episodes (at t+1) were selected. All listed items were selected as idiographic predictors of binge eating, independent of their significance. However, we additionally calculated the significance of the correlations for the context. The exact *P* values, codes, and data can be found in the corresponding project in the Open Science Framework [49]. Note that the results might vary slightly, as the R function *set.seed* does not apply to the cross tables.

#### Prediction of Binge Eating by Idiographic Predictor Subsets

The selection of idiographic predictor sets resulted in good average prediction accuracy (mean AUC 0.80, SD 0.15; mean 95% CI 0.63-0.95; mean specificity 0.87, SD 0.08; mean sensitivity 0.79, SD 0.19; mean maximum reliability of *r*_D_ 0.40, SD 0.13; mean *r*_CV_ 0.13, SD 0.31). The mean AUC of 0.80 indicates that there is on average an 80% chance that the idiographic models predict binge and nonbinge episodes accurately. The mean specificity of 0.87 indicates that the idiographic models mistakenly classified 13 of 100 episodes as binge-eating episodes. The mean sensitivity of 0.79 indicates that the idiographic models mistakenly classified 21 out of 100 binge-eating episodes as nonbinge episodes. Table 1 shows the prediction accuracies of the idiographic predictor subsets for binge-eating episodes per participant. R code and data are available from the Open Science Framework [49].

**Table 1:** Model fit indices for prediction of binge eating in the next 2.5 hours from idiographic predictors, selected by the Best Items Scale that is Cross-validated, Unit-weighted, Informative, and Transparent (BISCUIT) algorithm, separately for each participant.

		Participants												
		01	02	03	04	05	06	07	08	09	10	11	12	13
**Model fit indices**														
	AUC^a^ (95% CI)^b^	0.92 (0.75-1.00)	0.97 (0.92-1.00)	0.84 (0.70-0.98)	0.51 (0.23-0.80)	0.73 (0.45-1.00)	0.93 (0.77-1.00)	0.93 (0.83-1.00)	0.85 (0.75-0.95)	0.63 (0.41-0.85)	0.75 (0.58-0.93)	0.72 (0.53-0.92)	0.60 (0.29-0.90)	0.98 (0.94-1.00)
	Specificity	0.84	0.89	0.74	1.00	0.86	0.85	0.90	0.81	1.00	0.74	0.87	0.91	0.96
	Sensitivity	1.00	1.00	.86	.45	.67	1.00	1.00	.80	.56	.73	.69	.56	1.00
**Derivation step (within-sample performance)**														
	*r*_D_^c,d^ (SD)	0.48 (0.04)	0.42 (0.05)	0.53 (0.04)	0.33 (0.05)	0.34 (0.03)	0.47 (0.06)	0.53 (0.06)	0.51 (0.10)	0.10 (0.22)	0.46 (0.06)	0.50 (0.06)	0.18 (0.10)	0.32 (0.23)
**Validation step (out of sample performance)**														
	*r*_CV_^d,e^ (SD)	0.41 (0.34)	0.10 (0.56)	–0.02 (0.72)	0.36 (0.38)	0.38 (0.62)	–0.36 (0.28)	0.29 (0.30)	0.54 (0.42)	–0.11 (0.46)	0.34 (0.27)	0.27 (0.64)	0.08 (0.78)	–0.56 (0.57)

^a^AUC: area under the curve.

^b^CI: bootstrapped 95% CI of the AUC.

^c^*r*_D_: multiple *R* of the unit-weighted scale in the derivation step.

^d^*r*: pairwise Pearson correlation of the item with time-shifted binge-eating episodes (at t+1).

^e^*r*_CV_: average cross-validated multiple *R* of the derived scale.

## Discussion

### Principal Findings

#### Study 1—EMA-Item Set

This study used a mixed methods approach to develop a conceptual and statistical basis for an idiographic JITAI for binge eating. The EMA-item development in study 1 followed a replicable procedure similar to Soyster and Fisher [29] while considering nomothetic theories on binge-eating antecedents (ie, emotional eating) and underwent several qualitative (literature research and focus group brainstorming and discussion) and quantitative (focus group ratings) iterations and piloting.

This resulted in a broad EMA-item set (Multimedia Appendix 4), including several constructs underrepresented in the nomothetic literature (eg, “I feel detached from myself.”, and “specific” restrictions *“*did you restrict yourself [eg, by eating less, avoiding certain foods]?*”* [12,50,51]). This approach also helped us shorten the extensive lists of emotional states (eg, “right now I feel...calm/relived/ashamed/guilty/frustrated.”) because within-person ratings for similar emotions were often identical, and concerns about redundancy were expressed during the moderated discussion. Furthermore, we did not only incorporate risk factors into the EMA-item set but also protective factors that could potentially decrease the likelihood of binge eating (ie, healthy coping strategies to keep oneself from binge eating or positive emotions [27,28]). The role of protective factors is often overlooked in nomothetic binge-eating theories but is crucial to idiographic binge-eating prediction and intervention models.

#### Study 2—Prediction Based on Idiographic Predictor Subsets

Regularly completing extensive EMA-item sets (such as the present one with 36 interval-contingent and 20 event-contingent items) becomes increasingly burdensome over prolonged study periods. Thus, we applied a machine learning algorithm to the EMA data of patients with BN and BED to select parsimonious idiographic subsets of EMA items. This data-driven selection optimizes the predictive power within participants and decreases potential researcher bias.

The idiographic item subsets predicted binge-eating episodes with a high average accuracy (mean AUC 0.80) across 13 patients. Notably, the sensitivity approached 100% (successful prediction of every reported binge) in several patients, without forfeiting much specificity (predicting no binge when none occurred). This is noteworthy as outcome frequency was not extremely high (mean 10.4, SD 7.4; range 2-28 binge-eating episodes; see also Multimedia Appendix 6, Table S1 “Number of binge-eating episodes and total data points per participant”).

### Secondary Findings

Regarding the composition of the selected item sets, a high selection rate of items with high proximity to the binge-eating construct was evident (ie, hunger, food craving, urge to overeat, subjective binge-eating risk, and preceding binge-eating episodes). This suggests that some patients may accurately predict upcoming binge-eating episodes. This reveals a relatively high level of insight into the temporal evolution of the symptoms in some patients. Surprisingly, hunger and food craving were negatively correlated with binge eating in 3 patients. One could speculate that the negative correlations between hunger and binge eating in patients 06 and 11 point to disinhibition, that is, because of the temporary abandonment of rigid diet rules after eating in the absence of hunger [52,53].

In addition to items with conceptual similarity to binge eating, emotional items were selected in 9 patients. This supports the relevance of emotional eating in binge-eating predictions [4,11,19]. However, the selected emotion sets were highly heterogeneous across the 9 patients. In fact, no single emotion item (or specific set of emotion items) was consistently selected across all patients. This speaks against a singular and generalizable emotional eating theory of binge eating. Similarly, because no other nonemotion–related predictor was consistently selected across all patients, our pilot data provide no evidence for other generalizable nomothetic theories of binge eating. Thus, several nomothetic theories are needed to explain present heterogeneity, which may in turn explain the multitude of competing nomothetic binge-eating theories. Clearly, nomothetic theories must model individual differences more explicitly to account for these findings. These findings also support the use of a broad EMA-item set that covers a large range of possible binge-eating antecedents in the context of idiographic prediction [4-6,14-21].

Interestingly, time-derived predictors were selected only in 7 patients. In 6 of these patients, discrete time predictors were chosen that were consistent with the literature on the timing of binge-eating peaks (ie, afternoon to late evening) [43-45]. Time cycles were only selected in 3 patients. This is surprising given the observation of cyclic symptoms (eg, in depression [41]). However, time-based predictors may be more powerful if EMA items with conceptual similarity to binge eating are omitted. In the case of binge eating, time cycles could represent a rising and falling urge to overeat (eg, due to prolonged restriction between meals) [41,54]. Discrete time variables could represent the time of the day where a patient usually binges (eg, due to contexts such as being alone at home every afternoon) [41]. Assessing such time-derived variables does not require user input and thus does not contribute to the participant burden. This makes them valuable for the predictions in the JITAI framework.

### Limitations and Strengths

Compared with the high average within-sample performance (mean *r*_D_ 0.40), the average out-of-sample performance (mean *r*_CV_ 0.13) was lower, suggesting limited out-of-sample generalizability. This might be because of 10-fold cross-validation, which does not account for the serial correlation and potential nonstationarity of time-series data [55]. Future studies could resort to alternative time-series–specific techniques (ie, roll forward cross-validation and out-of-sample evaluation) that ensure that training data always precede test data. However, X-fold cross-validation has been shown to outperform these techniques [55]. Furthermore, the number of observations was limited (max 84 per participant), leading to relatively small splits in the 10-fold cross-validation. Thus, there was a high possibility of randomly drawn training sets that were unrepresentative of the data set. The results from the validation step might also vary slightly, as the R function *set.seed* does not apply to the cross tables.

Another general drawback of the BISCUIT algorithm is that nonlinear trends and interaction effects among predictors are not considered. In addition, when applied under optimal conditions (ie, big data sets and no missing data), gold standard machine learning approaches, such as random forests [56] and XGBoost [57] in combination with super learners [58], calibrate better to the data. However, for typical EMA data sets, the conditions are rarely optimal for these algorithms. Missing data and a limited number of observations are typical features of high-burden EMA sampling schemes. However, BISCUIT was created to handle these problematic properties. BISCUIT outperformed random forest and elastic net approaches in other studies with smaller idiographic data sets and more missing data (Beck et al [59]: mean 57.4, SD 16.3; range 40-109 EMA observations; present data: mean 67.3, SD 13.4; range 43-84 EMA observations).

Finally, the idiographic approach used in this study precludes mechanistic and theoretical inferences about binge eating. Generally, machine learning algorithms are silent about the underlying mechanisms; instead, they tailor models are as close as possible to the given data and conditions. Thus, the present results are highly specific, for example, to the used “prediction interval” of 2.5 hours between predictors and outcome. This could be problematic as it has been shown that emotions and eating can influence each other at different time intervals [60].

### Implications and Future Directions

In addition to emphasizing the importance of a broad predictor set, the results have a direct implication for the JITAI and EMA methodology: participant burden in longer EMA sampling periods precludes the use of large EMA-item sets. Thus, such EMA studies might prune their large EMA-item sets after a “calibration period” by applying the described predictor-selection approach. Therefore, the participant burden is reduced, whereas accurate idiographic binge-eating predictions are retained. Such predictions can then be used to trigger JITAIs, as done by Forman et al [24,61] in a JITAI on dietary lapses.

Future studies may transfer the present work to a range of disordered and maladaptive eating behaviors (eg, purging behaviors or food restrictions) to develop low-threshold JITAIs. EMA-item–based prediction should be compared with predictions generated from passive data sources (ie, smartphone sensors, use data, and wearable data) that do not inflict much user burden [10,62-64]. In the long term, acceptance, dropout rates, and effectiveness of JITAI protocols on binge eating need to be tested in microrandomized trials [65] and classic randomized controlled trials against nonadaptive, non–real-time interventions before the ultimate recommendation as the gold standard.

Finally, feeding back personal binge-eating predictors can serve as a psychoeducational intervention and raise awareness of personal risk and protective factors. Such personal binge-eating predictors can also inform conventional face-to-face psychotherapy. Patients with a clear dominance of emotion-related predictors might profit from emotion-focused interventions [66] more than patients with a dominance of impulsive or craving-related predictors, who might profit more from impulse control intervention [67].

## Appendix

Multimedia Appendix 1 Attrition diagram.

Multimedia Appendix 2 First item list.

Multimedia Appendix 3 Iterations in the creation of the item set.

Multimedia Appendix 4 Final ecologic momentary assessment items.

Multimedia Appendix 5 CONSORT (Consolidated Standards of Reporting Trials) flow diagram.

Multimedia Appendix 6 Number of binge-eating episodes and ecologic momentary assessment observations per patient.

## Abbreviations

AUC: area under the curve
BED: binge-eating disorder
BISCUIT: Best Item Scales that are Cross-validated, Unit-weighted, Informative and Transparent
BN: bulimia nervosa
CONSORT: Consolidated Standards of Reporting Trials
DSM-5: Diagnostic Statistical Manual-5
ED: eating disorder
EMA: ecologic momentary assessment
JITAI: just-in-time adaptive intervention
