# Supplementary material for: Toward Individualized Prediction of Binge-Eating Episodes Based on Ecological Momentary Assessment Data: Item Development and Pilot Study in Patients With Bulimia Nervosa and Binge-Eating Disorder
Source: JMIR Med Inform. 2023 Feb 23;11:e41513. doi: 10.2196/41513 (PMC9999257; doi:10.2196/41513)
Supplement: Multimedia Appendix 1 [file medinform_v11i1e41513_app1.docx]

## Multimedia Appendix 1

**Figure S1.**

Attrition diagram for literature research and review that lead to the first list of ecological momentary assessment antecedents of binge eating.

**509 articles identified**

(with the word “binge” in their title and the terms “ecological momentary” or “experience sampling” in any part of the article)

**262 articles retained**
(empirical EMA studies on binge eating)

First scan for relevance and duplicates

Global vector word embedding analysis of abstracts

**247 articles rejected**

- not relevant
- duplicates

**47 constructs retained** (for focus group etc.)

Ratings of detected constructs by two researchers
(quantifiable?)

**Rejected constructs**

- not quantifiable
- redundant
