# Supplementary material for: Toward Individualized Prediction of Binge-Eating Episodes Based on Ecological Momentary Assessment Data: Item Development and Pilot Study in Patients With Bulimia Nervosa and Binge-Eating Disorder
Source: JMIR Med Inform. 2023 Feb 23;11:e41513. doi: 10.2196/41513 (PMC9999257; doi:10.2196/41513)
Supplement: Multimedia Appendix 2 [file medinform_v11i1e41513_app2.docx]

## Multimedia Appendix 2

| Table S1.  *First set of EMA items and the ratings of their relation to binge-eating episodes (‘… happens before/during/after binge eating…’: 1 = (almost) never, 3 = might or might not, 5 = (almost) always) in a focus group of 11 patients with binge-eating episodes.* | | | |
| --- | --- | --- | --- |
| EMA item | *M* | *SD* | *Range* |
| Emotions |  |  |  |
| “Right now I feel...”  [„Ich fühle mich gerade…”] |  |  |  |
| tense [angespannt] | 4.91 | 0.30 | (4 – 5) |
| depressed [deprimiert] | 4.73 | 0.47 | (4 – 5) |
| frustrated [frustriert] | 4.73 | 0.47 | (4 – 5) |
| dissatisfied with myself [unzufrieden mit mir selbst] | 4.64 | 0.67 | (3 – 5) |
| ashamed [beschämt] | 4.64 | 0.67 | (3 – 5) |
| guilty [schuldig] | 4.64 | 0.67 | (3 – 5) |
| stressed [gestresst] | 4.45 | 0.52 | (4 – 5) |
| irritated [gereizt] | 4.36 | 0.81 | (3 – 5) |
| lonely [einsam] | 4.09 | 0.94 | (2 – 5) |
| worried [besorgt] | 3.82 | 1.17 | (1 – 5) |
| nervous [nervös] | 3.73 | 0.91 | (2 – 5) |
| bored [gelangweilt] | 2.91 | 1.38 | (1 – 5) |
| relived [erleichtert] | 1.91 | 1.14 | (1 – 4) |
| cheerful [fröhlich] | 1.20 | 0.42 | (1 – 2) |
| enthusiastic [begeistert] | 1.18 | 0.41 | (1 – 2) |
| relaxed [entspannt] | 1.18 | 0.41 | (1 – 2) |
| calm [gelassen] | 1.09 | 0.30 | (1 – 2) |
| Stressors |  |  |  |
| “I feel like I am not in control over everything.”  [„Ich habe das Gefühl, nicht alles im Griff zu haben.“] | 4.91 | 0.30 | (4 – 5) |
| “I feel strained due to…”  [„Ich fühle mich belastet von…“] |  |  |  |
| everyday stressors [allgemeinen Stressoren] | 4.82 | 0.41 | (4 – 5) |
| close social network  [engerem sozialem Umfeld] | 4.18 | 1.08 | (2 – 5) |
| wider social network  [weiterem sozialen Umfeld] | 3.50 | 1.27 | (1 – 5) |
| work/university/school [Arbeit/Universität/Schule] | 3.40 | 0.97 | (2 – 5) |
| Hunger |  |  |  |
| “I am dissatisfied with the amount of food that I consumed.” [„Ich bin unzufrieden mit der Nahrungsmenge, die ich bisher gegessen habe.“] | 4.00 | 1.34 | (1 – 5) |
| “Right now I am hungry.” [„Ich habe jetzt gerade Hunger.“] | 2.45 | 0.93 | (1 – 4) |

| Food craving |  |  |  |
| --- | --- | --- | --- |
| “I tried to resist my food craving.” [„Ich habe versucht, dem Verlangen nach verlockenden Nahrungsmitteln zu widerstehen.“] | 4.27 | 1.19 | (1 – 5) |
| “I feel like eating something tasty.”  [„Ich habe Lust, etwas Schmackhaftes zu essen.“] | 4.09 | 1.38 | (1 – 5) |
| “I had difficulties to resist eating tasty food.” [„Ich konnte schlecht auf den Konsum verlockender Nahrungsmittel verzichten.“] | 3.91 | 1.38 | (1 – 5) |
| Sport |  |  |  |
| “I engaged in increased levels of sports.” [„Ich habe vermehrt Sport getrieben.“] | 2.82 | 1.54 | (1 – 5) |
| Tiredness & exhaustion |  |  |  |
| “Right now I feel…” [„Ich fühle mich gerade…“] |  |  |  |
| exhausted [erschöpft] | 3.64 | 1.43 | (1 – 5) |
| tired [müde] | 3.27 | 1.49 | (1 – 5) |
| Other situations, circumstances and experiences |  |  |  |
| “I am on my own.” [„Ich bin alleine.“] | 4.73 | 0.65 | (3 – 5) |
| “I feel detached from myself.” [„Ich fühle mich nicht in Kontakt mit mir selbst.“] | 4.36 | 0.51 | (4 – 5) |
| “I just had an unpleasant contact with someone else.” [„Ich hatte gerade einen unnagenehmen Kontakt mit einem anderen Menschen.“] | 3.09 | 1.38 | (1 – 5) |
| “Right now I want to smoke a cigarette.“ [„Ich habe jetzt gerade Lust, Zigaretten zu rauchen.“] | 3.00 | 1.48 | (1 – 5) |
| “I just have drunken alcohol.” [„Ich habe gerade Alkohol getrunken.“] | 2.45 | 1.37 | (1 – 4) |
| “Right now I am shopping for groceries.” [„Ich bin gerade einkaufen.“] | 2.45 | 1.44 | (1 – 5) |
| “I bit my nails.” [„Ich habe an meinen Fingernägeln gekaut.“] | 2.36 | 1.80 | (1 – 5) |
| “I am in company.” [„Ich bin in Gesellschaft.“] | 2.27 | 1.01 | (1 – 4) |
| “I just smoked a cigarette.” [„Ich habe gerade geraucht.“] | 1.91 | 1.30 | (1 – 4) |
| Thoughts and behavior regarding food, weight and body shape |  |  |  |
| “I consciously ate less to influence my weight.” [„Ich habe bewusst weniger gegessen, um mein Gewicht zu beeinflussen.“] | 4.55 | 1.04 | (2 – 5) |
| “I was dissatisfied with my body weight and/or shape.” [„Ich war mit meinem Körpergewicht und/oder meiner Figur unzufrieden.“] | 4.55 | 1.04 | (2 – 5) |
| “Today, I was thinking about my body weight and/or shape.” [„Heute habe ich über Körpergewicht und/oder Figur nachgedacht.“] | 4.36 | 1.03 | (2 – 5) |
| “Today, I was often thinking about food/calories.” [„Heute habe ich häufig über Essen/Kalorien nachgedacht.“] | 4.27 | 1.27 | (1 – 5) |
| “I checked my body (e.g., by feeling/looking at parts of the body or weighting).” [„Ich habe meinen Körper kontrolliert (z.B. Abtasten/Betrachten von Körperpartien oder Wiegen).“] | 3.90 | 1.45 | (1 – 5) |
| “Regarding eating, I acted in the moment.” [„Ich habe beim Essen aus dem Moment heraus gehandelt.“] | 3.64 | 1.21 | (2 – 5) |
| “Today, I actively regulated my eating behavior according to my dietary goals.” [„Ich habe heute mein Essverhalten aktiv in Richtung meines Ernährungsziels gesteuert.“] | 2.91 | 1.70 | (1 – 5) |
| “I acted upon my plans regarding my eating behavior.” [„Ich bin beim Essen meinen Plänen/Vorsätzen gefolgt.“] | 2.45 | 1.51 | (1 – 5) |
