# Supplementary material for: Toward Individualized Prediction of Binge-Eating Episodes Based on Ecological Momentary Assessment Data: Item Development and Pilot Study in Patients With Bulimia Nervosa and Binge-Eating Disorder
Source: JMIR Med Inform. 2023 Feb 23;11:e41513. doi: 10.2196/41513 (PMC9999257; doi:10.2196/41513)
Supplement: Multimedia Appendix 3 [file medinform_v11i1e41513_app3.docx]

## Multimedia Appendix 3

**Figure S1.**

*Flowchart of the iterations in the creation of the final EMA item set.*

Clinicians
input

Literature research
and analysis

Focus
group

Researcher
input

First
pilot

Final
synthesis

Final item set

**4 added (eating based on internal opposed to external motivations; (not) following a regular meal structure; contact with beauty ideals in media; currently restricting on certain foods (*later cut*))**

**2 merged to 1 (“I am in company.” and “I am on my own.” 🡪 “Are you on your own right now?”)**

**4 rephrased to 5 (“I tried to resist my food craving.” 🡪 “How much did you try to resist your craving for tempting food since the last entry?”;“I had difficulties to resist my food craving.” 🡪 “How well did you resist your craving for tempting food since the last entry?” (Cut later);“I consciously ate less to influence my weight.” 🡪 “Did you restrict yourself (e.g., by eating less, avoiding certain foods)?”; “I just had an unpleasant contact with someone else.”-> “Have you been in contact to other people since the last entry?” and “How was your contact to other people since the last entry?”)**

**4 exchanged by 1 (“I feel strained due to…” work/university/school; close social network; wider social network; everyday stressors 🡪 “Do you feel like you can handle all upcoming tasks and problems?”)**

**6 added (“How was your sleep?”; “When did you fall asleep approximately?”; “When did you wake up today approximately?”; “How much do you want to regulate your eating behavior actively towards your dietary goals tomorrow?”); (*added but later cut again*) “Do you feel like smoking a cigarette right now?”;),**

**2 split into 4 (“Today, I was thinking about my body weight and/or shape.” 🡪 Was split into: “Did you often think about your body weight today?” and “Did you often think about your physique today?”; „I was dissatisfied with my body weight and/or shape.” 🡪 was split into: “How satisfied have you been with your body weight today?” and “How satisfied have you been with your physique today?”)**

**1 added („How much did you try to distract yourself from a possible urge to overeat by healthy strategies (e.g., relaxation, social activity, mindfulness, etc.)?”)**

**4 merged to 1 (“I bit my nails.”, “Right now I want to smoke a cigarette.“, “I just smoked a cigarette.”, and “I just drank alcohol.” 🡪 „How much did you try to distract yourself from a possible urge to overeat by unhealthy strategies (e.g., alcohol, cigarettes, drugs, self-harm, etc.)?”)**

**2 rephrased into 2 (“Today, I actively regulated my eating behavior according to my dietary goals.” 🡪 “How much did you try to regulate your eating behavior successfully today?”; “How much do you want to regulate your eating behavior actively towards your dietary goals tomorrow?” 🡪 “How much do you want try to regulate your eating behavior successfully tomorrow?”)**

**1 split into 3 (“I feel like eating something tasty.” (meant to embody the construct food craving) was split into multiple items to embody multiple constructs.🡪 Food craving: “How strong is your craving after certain foods right now?”; Overeating: “How strong is your urge to overeat right now?”; Binge eating episodes: “How high would you rate your risk for a binge-eating episode right now?”)**

**4 added ( “Did you experience unusual circumstances regarding food since the last entry (e.g., invitations, sales, limited variety)?”; “How typical was today for you in terms of everyday life?” (*added due to the ongoing COVID-19 pandemic*); “How much was your eating behavior influenced by unusual circumstances today (e.g., invitations, sales, limited variety)?”; “How much was your eating behavior influenced by emotions today?”)**

**1 rephrased (“Until the next entry it is likely that I will encounter tempting food (i.e., throughout shopping)” 🡪 „Were tasty foods available to you since the last entry?“)**

**47 items**

**39 items**

**53 items**

**48 items**

**52 items**

**52 items (interval contingent)**

**+20 items (event contingent)**

**1 rejected**(“Regarding eating, I acted in the moment.”)

**9 rejected
(tiered; calm; relived; ashamed; guilty; frustrated Right now I am shopping for groceries; I acted upon my plans regarding my eating behavior; I engaged in increased levels of sport)**

**5 rejected
(“Are you restricting on certain foods right now?“; “Do you feel like smoking a cigarette right now?”; “Until the next entry it is likely that external circumstances will prevent me from healthy eating behavior (i.e., invitations, no availability of healthy food)”, “… I will feel unable to eat according to my dietary goals.”; “How well did you resist your craving for tempting food since the last entry?”**

**Interval contingent questionnaire (see Multimedia Appendix 4: Table S1):**

- - **every questionnaire 36 items**
  - in the morning (+3 items)
  - in the evening (+13 items)

**Event questionnaire on overeating & binge eating: 20 items (see Multimedia Appendix 4: Table S2)**

**First list (see Multimedia Appendix 2: Table A1)**

**46 items**

**7 added (“Until the next entry it is likely that… I will encounter tempting food (i.e., throughout shopping)”; "… external circumstances will prevent me from healthy eating behavior (i.e., invitations, no availability of healthy food)“; "… I will feel unable to eat according to my dietary goals.“ (*later rephrased*); “Did you eat something since the last entry?”; “How much did you eat?”; “Was your meal a… (Main meal, Snack, Binge)”; “Did you eat another meal?”); (no additional item, but idea to add the autoregressive effect of binge-eating episodes as predictor)**

**2 rephrased to 2 (“Are you on your own right now?” 🡪 “Did you eat alone or in company?”; “I feel like eating something tasty.” -> „How strong is your craving for certain, high caloric, tasty foods right now?“ (*later rephrased again*))**
