# Supplementary material for: Toward Individualized Prediction of Binge-Eating Episodes Based on Ecological Momentary Assessment Data: Item Development and Pilot Study in Patients With Bulimia Nervosa and Binge-Eating Disorder
Source: JMIR Med Inform. 2023 Feb 23;11:e41513. doi: 10.2196/41513 (PMC9999257; doi:10.2196/41513)
Supplement: Multimedia Appendix 4 [file medinform_v11i1e41513_app4.docx]

## Multimedia Appendix 4

| Table S1.  *Final set of signal-contingent EMA items.* |
| --- |
| Final signal-contingent EMA item set |
| *Only at the first EMA questionnaire in the morning* |
| Sleep |
| “How was your sleep?” [„Wie war Ihr Schlaf?”] |
| “When did you fall asleep approximately?” [„Wann schliefen Sie in etwa ein?”] |
| “When did you wake up today approximately?” [„Wann sind Sie morgens in etwa aufgewacht?”] |
| *At every EMA questionnaire* |
| Hunger, craving, overeating and risk to binge |
| “Are you hungry right now?” [„Haben Sie jetzt gerade Hunger?“] |
| “How strong is your craving for certain foods right now?” [„Wie stark ist jetzt gerade Ihr Verlangen, bestimmte Nahrungsmittel zu essen?“] |
| “How strong is your urge to overeat right now?”  [ „Wie stark ist Ihr derzeitiger Drang sich zu überessen?“] |
| “How high would you rate your risk for a binge-eating episode right now?” [ „Wie hoch schätzen Sie Ihr derzeitiges Risiko für einen Essanfall ein?“] |
| Emotions |
| “How do you feel right now...?” [„Wie fühlen Sie sich im Moment…”] |
| Cheerful [fröhlich] |
| Enthusiastic [begeistert] |
| Relaxed [entspannt] |
| Irritated [gereizt] |
| Worried [besorgt] |
| Depressed [deprimiert] |
| Bored [gelangweilt] |
| Nervous [nervös] |
| Dissatisfied with myself [unzufrieden mit mir selbst] |
| Tense [angespannt] |
| Stressed [gestresst] |
| Lonely [einsam] |
| Stressors |
| “Do you feel in control?” [„Haben Sie das Gefühl, alles im Griff zu haben?“] |
| “Do you feel like you can handle all upcoming tasks and problems?”  [„Haben Sie das Gefühl, mit allen anstehenden Aufgaben und Problemen richtig umgehen zu können?“] |
| Exhaustion |
| “How exhausted are you feeling right now?” [„Wie erschöpft fühlen Sie sich gerade?“] |

| Other circumstances and experiences |
| --- |
| “How much are you feeling in touch with yourself right now?”  [„Wie sehr fühlen Sie sich gerade in Kontakt sich selbst?“] |
| “Are you on your own right now?” [„Sind Sie gerade alleine?“] |
| Resistance |
| “Were tempting foods available to you since the last entry?” [„Waren seit der letzten Eingabe verlockende Nahrungsmitteln für Sie verfügbar?“] |
| “How much did you try to resist your craving for tasty food since the last entry?” [„Wie sehr haben Sie seit der letzten Eingabe versucht, dem Verlangen nach verlockenden Nahrungsmitteln zu widerstehen?“] |
| Eating episodes |
| “Did you eat something since the last entry?” [“Haben Sie seit der letzten Eingabe etwas gegessen?“] |
| “Did you eat alone or in company?” [“Haben Sie allein oder in Gesellschaft gegessen?“] |
| “How much did you eat?” [„Wieviel haben Sie gegessen?“] |
| “How satisfied were you with that amount of food?” [„Wie zufrieden waren Sie mit dieser Nahrungsmenge?“] |
| “Did you restrict yourself (e.g., by eating less, avoiding certain foods)?” [„Haben Sie sich bestimmten Einschränkungen unterzogen (z.B. geringere Mengen, Vermeidung von bestimmten Lebensmitteln)?“] |
| “Was your meal a…” [„War Ihre Mahlzeit ein/e…“] |
| Main meal [Hauptmahlzeit] |
| Snack [Zwischenmahlzeit] |
| Binge [Essanfall] |
| “Did you eat on your own accord?” (Did you have food sovereignty?) [“Haben Sie aus eigenem Antrieb gegessen?“] |
| “Did you eat another meal?” [„Haben Sie noch etwas gegessen?“] |
| Yes [Ja] |
| No [Nein] |
| “Have you been in contact with other people since the last entry?” [„Waren Sie seit der letzten Eingabe in Kontakt mit anderen Personen?“] |
| “How was your contact with other people since the last entry?” [„Wie war Ihr Kontakt mit anderen Personen seit der letzten Eingabe?“] |
| **Distraction and compensation** |
| **„How much did you try to distract yourself from a possible urge to overeat by *unhealthy* strategies (e.g., alcohol, cigarettes, drugs, self-harm, etc.)?”** [„Wie sehr haben Sie sich seit der letzten Eingabe durch *ungesunde* Strategien von einem möglichen Drang zu überessen abgelenkt (z.B. Alkohol, Zigaretten, Drogen, Selbstverletzung)?“] |
| **„How much did you try to distract yourself from a possible urge to overeat by *healthy* strategies (e.g., relaxation, social activity, mindfulness, etc.)?”** [„Wie sehr haben Sie sich seit der letzten Eingabe durch *gesunde* Strategien von einem möglichen Drang zu überessen abgelenkt (z.B. Entspannung, soziale Aktivität, Achtsamkeit, etc.)?“] |
| “Did you experience unusual circumstances regarding food since the last entry (e.g., social eating, food on sale, restricted food supply)?” [„Gab es seit der letzten Eingabe ungewöhnliche Umstände bezüglich Essen (z.B. Einladungen, Angebote, eingeschränkte Auswahl)?“] |
| *Only at the last EMA questionnaire in the evening* |
| “How typical was today for you in terms of everyday life?” [„Wie alltagstypisch war der heutige Tag für Sie?“] |
| Successful regulation of eating behavior |
| “How much did you try to regulate your eating behavior successfully *today*?”  [„Wie sehr haben Sie *heute* versucht Ihr Essverhalten erfolgreich zu steuern?“] |
| “How much do you want to try to regulate your eating behavior successfully *tomorrow*?” [„Wie sehr wollen Sie *morgen* Ihr Essverhalten erfolgreich steuern?“] |
| „How much did you follow a regular meal structure today?” [„Wie sehr haben Sie heute eine regelmäßige Mahlzeitenstruktur eingehalten?“] |
| Food, body weight and shape |
| “Did you often think about food / calories today?” [„Haben Sie heute häufig über Essen / Kalorien nachgedacht?“] |
| “Did you often think about your body weight today?” [„Haben Sie heute häufig über Ihr Körpergewicht nachgedacht?“] |
| “How satisfied have you been with your body weight today?” [„Wie zufrieden waren Sie heute mit Ihrem Körpergewicht?“] |
| “Did you often think about your physique today?” [„Haben Sie heute häufig über Ihre Figur nachgedacht?“] |
| “How satisfied have you been with your physique today?” [„Wie zufrieden waren Sie heute mit Ihrer Figur?“] |
| “Have you ‘controlled’ your body today (e.g., by feeling your body / inspecting parts of your body or weighting)?” [„Haben Sie heute Ihren Körper „kontrolliert“ (z.B. durch Abtasten / Betrachten von Körperpartien im Spiegel oder Wiegen)?“] |
| Other |
| “Did you engage in media displaying beauty ideals today?” [„Haben Sie sich heute mit Medien beschäftigt, die Schönheitsideale dargestellt haben?“] |
| “How much was your eating behavior influenced by unusual circumstances today (e.g., invitations, sales, limited variety)?” [„Wie sehr haben heute ungewöhnliche Umstände (z.B. Einladungen, Angebote, eingeschränkte Auswahl) Ihr Essverhalten beeinflusst?“] |
| “How much was your eating behavior influenced by emotions today?” [„Wie sehr haben heute Emotionen Ihr Essverhalten beeinflusst?“] |

*Note.* The final EMA item set assessed all items listed in Table A2 and A3. The prediction algorithm only processed the items in black. We decided to process only these items by the algorithm as the other items were assessed only once per day (thus in the best case, if all EMA questionnaires are answered, they would be answered only 14 times within individual participants, less or never in most participants). The items regarding eating episodes were averaged per questionnaire within participant whenever more than one eating episode was reported in one questionnaire. Thus the question regarding ‘eating alone or in company’ was excluded as this cannot be averaged.

**Table S2.**
*Final set of event-contingent EMA items.*

| **Final event-contingent EMA item set** |
| --- |
| *Participants were instructed to open the event-contingent questionnaire only if they experienced an episode of subjective overeating, objective overeating or binge eating. The terms were explained to every participant by a trained graduate-student level researcher.* |
| **Emotions** |
| **“How do you feel right now...?”** [„Wie fühlen Sie sich im Moment…”] |
| **Cheerful** [fröhlich] |
| **Enthusiastic** [begeistert] |
| **Relaxed** [entspannt] |
| **Relieved** [erleichtert] |
| **Irritated** [gereizt] |
| **Worried** [besorgt] |
| **Depressed** [deprimiert] |
| **Bored** [gelangweilt] |
| **Nervous** [nervös] |
| **Dissatisfied with myself** [unzufrieden mit mir selbst] |
| **Tense** [angespannt] |
| **Stressed** [gestresst] |
| **Lonely** [einsam] |
| **Ashamed** [beschämt] |
| **Guilty** [schuldig] |
| **Classification: subjective or objective overeating or binge eating** |
| **“Would you rate the amount of food as excessive?”**  [„Sehen Sie die Essensmenge als übermäßig an?“] |
| **“Would other people rate the amount of food as excessive (under similar circumstances)?”** [„Würden andere Menschen die Essensmenge als ungewöhnlich groß ansehen (unter ähnlichen Umständen)?“] |
| **“Did you feel like you are losing control of your eating behavior?”**  [„Hatten Sie das Gefühl, die Kontrolle über das Essen zu verlieren?”] |
| **Compensatory behavior** |
| **“Did you engage in compensatory behavior?”**  [„Haben Sie eine Kompensationsmaßnahme angewendet?“] |
| **Self-induced vomiting** [selbstinduziertes Erbrechen] |
| **Consumption of diuretics or laxatives** [Einnahme von Abführ- oder Entwässerungsmitteln] |
| **Skipping one or multiple meals** [Auslassen einer oder mehrerer Mahlzeiten] |
| **Increased physical activity** [Vermehrtes Sporttreiben] |
| **Other: free text field** [Sonstiges: freies Textfeld] |
| **“Do you plan to engage in compensatory behavior?”**  [„Planen Sie eine Kompensationsmaßnahme anzuwenden?“] |
| **Self-induced vomiting** [selbstinduziertes Erbrechen] |
| **Consumption of diuretics or laxatives** [Einnahme von Abführ- oder Entwässerungsmitteln] |
| **Skipping one or multiple meals** [Auslassen einer oder mehrerer Mahlzeiten] |
| **Increased physical activity** [Vermehrtes Sporttreiben] |
| **Other: free text field** [Sonstiges: freies Textfeld] |
| **Self-licensing** |
| **“I overate because I was not able to build up enough resistance against my craving.”**  [„Ich habe mich übergessen, weil ich nicht genug Widerstand gegen mein Verlangen aufbauen konnte.”] |
| **“I overate because my thoughts (e.g., “Now it does not matter anymore”) made my give up my resistance.”**  [„Ich habe mich übergessen, weil mich meine Gedanken (z.B. "jetzt ist es auch egal") dazu brachten, meinen Widerstand aufzugeben.“] |

*Note.* The final EMA item set assessed all items listed in Table A2 and A3. The prediction algorithm only processed the items in black. We decided to process only these items by the algorithm as the other items were assessed only once per day (thus in the best case, if all EMA questionnaires are answered, they would be answered only 14 times within individual participants, less or never in most participants). From the event-contingent questionnaire in Table A3 only the classifier items for objective binge-eating were used for the algorithm.
