# Supplementary material for: Toward Individualized Prediction of Binge-Eating Episodes Based on Ecological Momentary Assessment Data: Item Development and Pilot Study in Patients With Bulimia Nervosa and Binge-Eating Disorder
Source: JMIR Med Inform. 2023 Feb 23;11:e41513. doi: 10.2196/41513 (PMC9999257; doi:10.2196/41513)
Supplement: Multimedia Appendix 5 [file medinform_v11i1e41513_app5.docx]

## Multimedia Appendix 5

**Figure S1**

Consort flow diagram.

**2 weeks just in time adaptive intervention (JITAI; *n*=17)**

**Interviewed for eligibility (*n*=25)**

**2 weeks ecological momentary assessment (EMA; *n*=22)**

**Excluded (*n*=3)**

- No BN and BED research diagnosis (*n*=2)
- Withdraw for participation (*n*=1)

**Follow-up (online questionnaire after 2 weeks; *n*=15)**

**Dropout (*n*=1)**

- Admission to inpatient treatment (*n*=1)

**Enrolment (*n*=22)**

**Dropout (*n*=9/*n*=4)**

- Admission to inpatient treatment (*n*=3)
- Technical issues (*n*=1)

*Only relevant for the present manuscript (NOT for the next study phase):*

- Not answering a minimum of 42 out of 84 EMA prompts (*n*=2)
- Did not report any objective binge-eating episodes 🡪 no outcome for analyzes (*n*=3)

**Dropout (*n*=2)**

- Did not answer the follow-up questionnaire (*n*=2)

**Analyzed in present manuscript (*n*=13)**

**Applicants (*n*=42)**

**Dropout (*n*=17)**

- Did not answer to make an interview appointment / withdraw for participation (*n*=13)
- No android smartphone (*n*=3)
- Admission to inpatient treatment (*n*=1)

**Completion rate EMA (18 / 22) = 0.82**

**Completion rate JITAI (17 / 22) = 0.77**

**Completion rate Follow-up (15 / 22) = 0.68**

The number of people completing the EMA phase (and subsequent JITAI phase, and follow-up), divided by the number of people who enrolled.
