# Supplementary material for: Toward Individualized Prediction of Binge-Eating Episodes Based on Ecological Momentary Assessment Data: Item Development and Pilot Study in Patients With Bulimia Nervosa and Binge-Eating Disorder
Source: JMIR Med Inform. 2023 Feb 23;11:e41513. doi: 10.2196/41513 (PMC9999257; doi:10.2196/41513)
Supplement: Multimedia Appendix 6 [file medinform_v11i1e41513_app6.docx]

## Multimedia Appendix 6

**Table S1.**

Number of binge-eating episodes and total data points per participant.

| **Participants** | | | | | | | | | | | | | | | |  |
| --- | --- | --- | --- | --- | --- | --- | --- | --- | --- | --- | --- | --- | --- | --- | --- | --- |
|  | **BN** | | | | | | | | | | | | | **BED** | |  |
|  | | 01 | 02 | 03 | 04 | 05 | 06 | 07 | 08 | 09 | 10 | 11 | 12 | | 13 | |
| **Percentage of answered EMA observations** | | 86% (72 of 84) | 64% (54 of 84) | 64% (54 of 84) | 81% (68 of 84) | 100% (84 of 84) | 51% (43 of 84) | 55% (46 of 84) | 96% (81 of 84) | 100% (84 of 84) | 92% (77 of 84) | 90% (76 of 84) | 79% (66 of 84) | | 83% (70 of 84) | |
| Binge-eating episodes reported by **EMA** (during the 2-week EMA phase | | 2 | 6 | 8 | 14 | 6 | 5 | 2 | 28 | 17 | 16 | 16 | 9 | | 6 | |
| Binge-eating episodes reported in retrospective **Binge eating online questionnaire** (not during EMA but directly after the 2-week EMA phase) | | 6 | 7 | 12 | 9 | 3 | 5 | 2 | 14 | 5 | 8 | 25 | 5 | | 4 | |
